# The Phenomenology of Specialization of Criminal Suspects

**DOI:** 10.1371/journal.pone.0064703

**Published:** 2013-05-15

**Authors:** Michele Tumminello, Christofer Edling, Fredrik Liljeros, Rosario N. Mantegna, Jerzy Sarnecki

**Affiliations:** 1 Department of Social and Decision Sciences, Carnegie Mellon University, Pittsburgh, Pennsylvania, United States of America; 2 Department of Statistical and Mathematical Sciences “Silvio Vianelli,” University of Palermo, Palermo, Italy; 3 Department of Sociology, Lund University, Lund, Sweden; 4 Institute for Futures Studies, Stockholm, Sweden; 5 Department of Sociology, Stockholm University, Stockholm, Sweden; 6 Department of Physics and Chemistry, University of Palermo, Palermo, Italy; 7 Center for Network Science and Department of Economics, Central European University, Budapest, Hungary; 8 Department of Criminology, Stockholm University, Stockholm, Sweden; Northwestern University, United States of America

## Abstract

A criminal career can be either general, with the criminal committing different types of crimes, or specialized, with the criminal committing a specific type of crime. A central problem in the study of crime specialization is to determine, from the perspective of the criminal, which crimes should be considered similar and which crimes should be considered distinct. We study a large set of Swedish suspects to empirically investigate generalist and specialist behavior in crime. We show that there is a large group of suspects who can be described as generalists. At the same time, we observe a non-trivial pattern of specialization across age and gender of suspects. Women are less prone to commit crimes of certain types, and, for instance, are more prone to specialize in crimes related to fraud. We also find evidence of temporal specialization of suspects. Older persons are more specialized than younger ones, and some crime types are preferentially committed by suspects of different ages.

## Introduction

Specialization in crime is a central problem for criminology as well as crime prevention and enforcement [1], [2]. Understanding specialization in crime implies the possibility of increasing the efficiency of the justice system through various measures targeting different groups of offenders (e.g., using selective detention and targeted treatment). The problem of specialization in crime is complex, both theoretically and methodologically. Although the empirical results that support the existence of crime specialization are admittedly weak, research has not yet been able to rule out specialization [3]. The issue is whether some offenders commit a disproportionately large number of the same type of crime during their criminal career, and whether this tendency is accentuated over time [4]. In this study we advance previous research on criminal careers by studying how different types of crimes are interrelated. This allows us to detect structural patterns in criminal behavior at the collective level that are not emergent at the individual level.

### Theoretical Aspects

Many criminologists [5]–[7] have noted that the existence of specialization (or lack thereof) has major implications for understanding the causes of crime. Gottfredson and Hirschi's influential *A General Theory of Crime* [8], for example, articulates positions that are inconsistent with the idea of crime specialization. According to their theory, persons with low self-control are expected to be more prone to criminal behavior than persons with high self-control. Among persons with low self-control, we should expect a great diversity of crime types [8]. Social-bond theory [9], [10], which focuses on the importance of attachment, commitment, and involvement in society along with pro-social beliefs, also suggests that offenders are generalists, who commit crimes due to a low level of social control.

Other research strongly points to specialization by focusing on the causes of specific types of crimes. Examples include theories targeting the relationship between the various functions of the brain and delinquency, such as brain damage [11], [12] and low or unstable serotonin levels [13], [14]. This line of research focuses primarily on the causes of violent crime, assuming that aggressive individuals are specialists in violent crime. Similar considerations apply to theories that search for causes of violence in the interaction between genetic and social factors [15], or in social rather than biological factors [16].

The main thesis in Sutherland's theory on differential association [17], [18] is that crime is a learned behavior, suggesting a high level of crime specialization. Sutherland proposed that learning about criminal behavior, much like learning about other behaviors, occurs through interactions with those in the individual's immediate social environment. The theory assumes that the individual's behavior is influenced by the total outcome of the influences received from the social environment. If the individual’s social environment is composed of a large number of individuals who are contemptuous of the law and break it with little compunction, the likelihood of delinquent behavior increases. Accordingly, we therefore expect specialization in crime within this theory.

It has also been suggested that specialization emerges during a criminal career. Cloward and Ohlin [19] argued that a lack of legitimate opportunities leads to three different kinds of delinquent subcultures that specialize in specific types of crimes, depending on the structure of the illegitimate opportunities.

### Methodological Problems

What constitutes a crime, and consequently criminal behavior, varies considerably across time and space [20]. This variability introduces some challenges in determining whether an individual is engaging in repetitive or diversified criminal behavior over the long term. Creating appropriate distinctions among different criminal acts is difficult. Legal classification offers a comprehensive breakdown into many small categories of crime organized based on chapters of the penal code. We take advantage of the finely detailed resolution of official classification and coding. In fact, the classification allows us to empirically establish how crime categories organize into larger clusters that emerge from individual’s criminal behavior.

Our database is the Swedish national register of persons suspected of criminal offenses, which contains more detailed information on crimes as compared to registers on sentenced persons. Due to the nature of records of suspected criminal offences, a proportion of all committed crimes is not covered, which is a limitation of our data. On the other hand, our data have three significant advantages over self-reported data. In fact, our database includes information about a very large number of crimes, precise information on the timing, and detailed legal definition of the offenses.

In this paper we apply methods from network analysis [21] to identify the systematic occurrence of crimes in a large set of criminal suspects. This is a new way of using network analytical tools in criminology. Network analysis has mainly been used to study co-offending [22], where individuals are nodes and offenses define edges. In this study, we do the reverse and treat types of crimes as nodes and individuals as defining the edges, allowing us to study the clustering of types of crimes.

## Materials and Methods

### Description of the Database

We have information about all the suspects of crimes committed in the Stockholm area during the period from 1991 to 2007, including a coded identity of suspects, their gender and age, and the types of crimes they have been suspected of. Crime in Sweden is on average of Western European level [23]. Stockholm is Sweden's capital and the country's largest city (870 000 inhabitants). Crime in Stockholm per 100,000 population is at slightly higher level compared to the rest of the country. N_C_ = 376 different types of crimes, according to the penal code, appear in the database. These types of crimes were attached to N = 336,069 different suspects. It is worth noting an essential aspect of these data. As in most complex systems, the data show a large degree of heterogeneity. In the investigated time period, 12 rare types of crimes occur only once, whereas, at the other extreme, one rather common type of crime was implicated to as many as 81,532 different suspects. In short, the range of the number of different suspects implicated in a given type of crime spans almost five orders of magnitude. Another source of heterogeneity lies in the number of different types of crimes each suspect has been suspected of. A total of 169,603 suspects were suspected of only a single type of crime and, at other extreme, one specific suspect has been implicated in 159 different types of crimes. So, even in terms of suspects, we observe heterogeneity over more than two orders of magnitude (see also Fig. S1). Different criminal instances are recorded for each type of crime. The total number of criminal instances is 1,851,960. The interval of criminal instances for suspect is ranging from the minimum value of 1 to the maximum value of 2,347.

### Spectral Analysis

We use spectral analysis to establish the extent to which there is a tendency toward generalist or specialist criminal behavior. Specifically, we first determine the correlation among types of crimes in the following way. The correlation matrix of types of crimes is calculated by associating a vector of dimension N = 336,069, that is the total number of suspects in the database, with each type of crime. For each type of crime C, the *C_i_* component of the associated vector is set 1 if the suspect *i* has been suspected of crime type C, or 0 otherwise. The correlation between two types of crimes A and B is calculated as the correlation coefficient between the corresponding vectors:
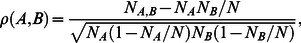
(1)where *N_A_* (*N_B_*) is the total number of suspects implicated in type of crime A (B), and *N_A,B_* the total number of suspects implicated in both types of crimes.

We then focus on the spectrum of eigenvalues of the correlation matrix of types of crimes, that is the matrix of correlation coefficients (1). The analysis of eigenvalues and eigenvectors of the correlation matrix allows us to see that the eigenvector of the largest eigenvalue presents most of its components of the same sign, and therefore does not present a block-like structure that would indicate a partitioning of different types of crimes. The existence of such a “common mode” [24], [25] in the present system can be interpreted as indicating generalist behavior in some suspects. Specifically, the largest eigenvalue of the sample correlation matrix is λ_M_ = 11.26, and 89% of the components of the corresponding eigenvector have the same sign. The presence of 169,603 people suspected of only a single type of crime is not altering the basic characteristics of the correlation matrix. In fact, by repeating the analysis with only suspects implicated in two or more types of crimes we obtain very similar results (see Fig. S2).

**Figure 1:**
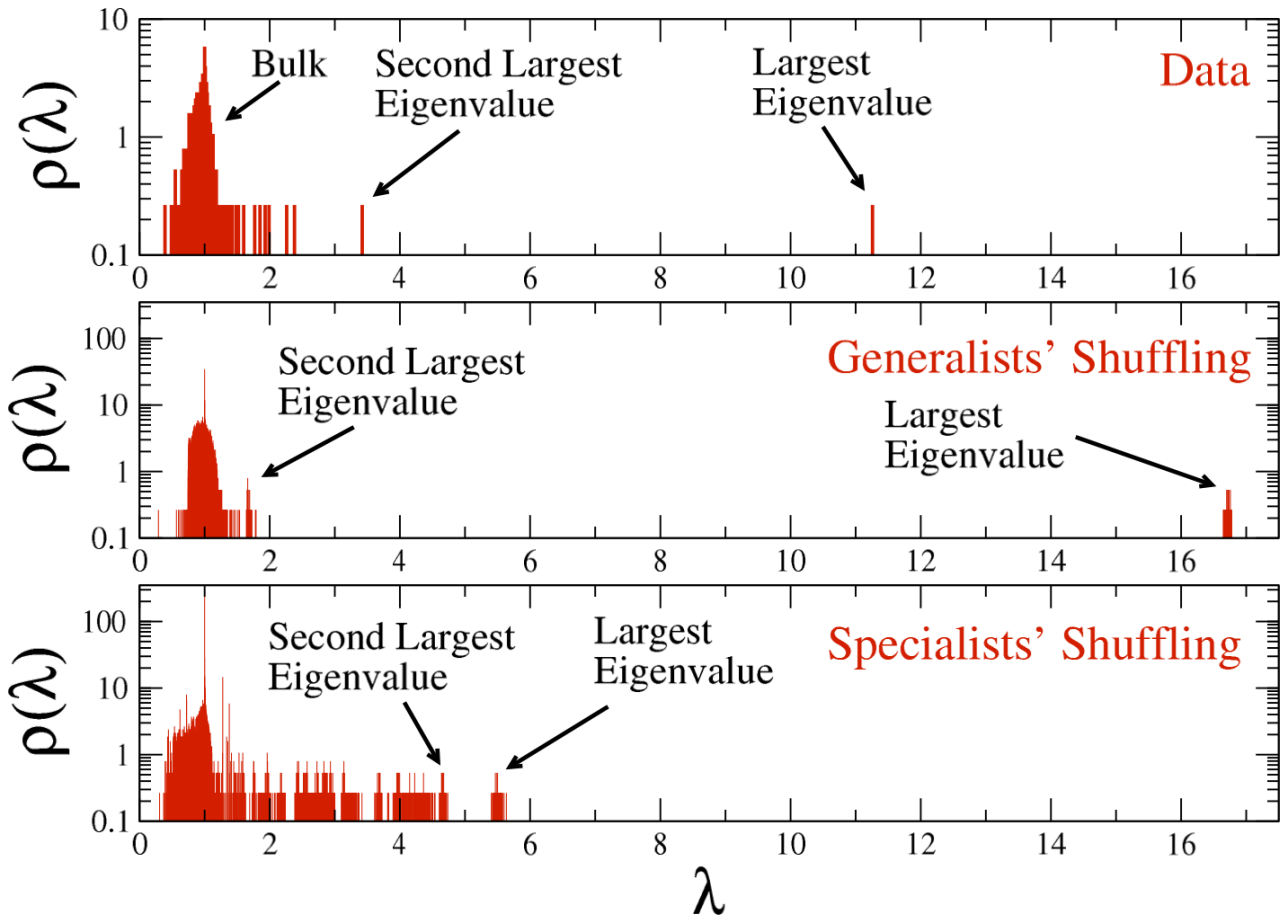
Density of eigenvalues of the correlation matrix of types of crimes associated with (i) the original data (top panel); (ii) 100 random shufflings of the database mimicking pure generalists (middle panel); and (iii) 100 random shufflings of the database mimicking pure specialists (bottom panel). The largest eigenvalue of the empirical correlation matrix, which accounts for the generalist behavior of suspects, is about two third of the corresponding eigenvalue in the case of pure generalists, and about two times the one obtained for pure specialists. The second largest eigenvalue of the empirical correlation matrix, which suggests the presence of clusters of types of crimes, and therefore the presence of specialists in the set of suspects, is also intermediate between the corresponding ones obtained for pure generalists and pure specialists.

**Figure 2:**
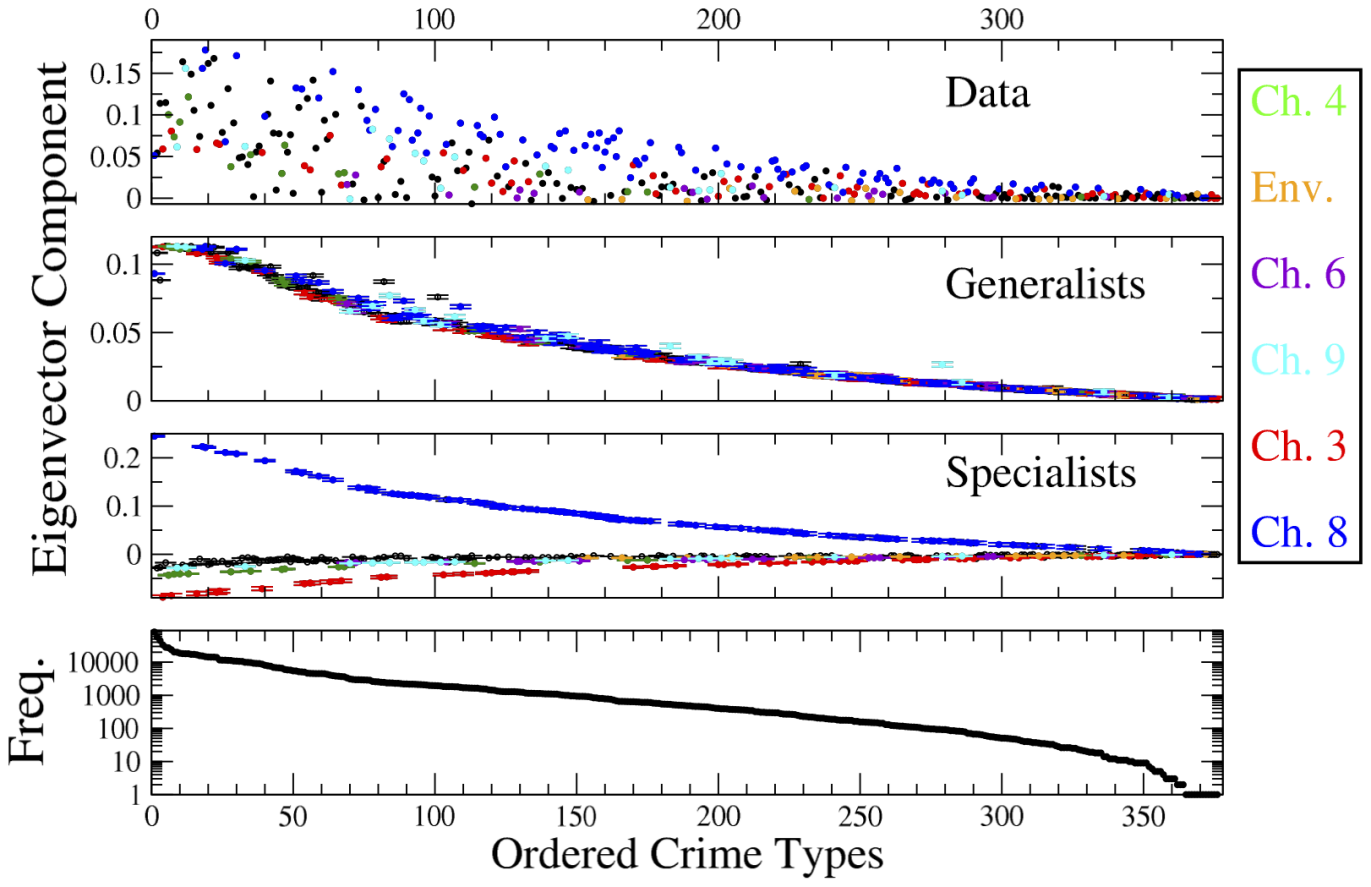
Components of the eigenvector corresponding to the first eigenvalue of the correlation matrix of types of crimes for the original data, and average components of the first eigenvector over 100 independent realizations of the generalists' and specialists' shufflings. In the latter cases, error bars correspond to one standard deviation over 100 realizations. The color of a spot indicates the chapter of the penal code of types of crimes belonging to the 6 most populated chapters. Specifically, we label types of crimes as follows: chapter 8 - theft, robbery and other types of crimes of stealing as blue (102 crimes), chapter 3 - types of crimes against life and health as red (60 crimes), chapter 9 - fraud and other acts of dishonesty as cyan (22 crimes), chapter 6 - sexual offences as violet (21 crimes), offences against the environmental code as orange (19 crimes), and chapter 4 - types of crimes against liberty and peace as green (17 crimes). In the case of generalists' and specialists' simulations we also show the interval of plus and minus one standard deviation around the average value. Types of crimes are ordered according to their occurrence in the database as shown in the bottom panel of the figure.

To provide a more quantitative indication of the presence of generalists in the database and their impact on the properties of the correlation matrix of types of crimes, we compare the empirical spectrum of the sample correlation matrix with the spectrum of a correlation matrix R_G_ obtained from a random shuffling of the original database. In the shuffling we preserve the heterogeneity of both suspects and types of crimes observed in the original data. Specific criminal patterns, i.e., specialist patterns within particular groups of types of crimes, are destroyed by the shuffling, and all the suspects therefore present a generalist profile in the shuffled realizations. Specifically, in our shuffling procedures we perform 100 different realizations. The largest eigenvalue of R_G_ has a mean value of 16.72 (see Fig. 1), which corresponds to 16.72/N_C_ = 0.045 of the total variance, and the corresponding eigenvector (see Fig. 2) essentially displays all components with the same sign.

The comparison between the top and middle panel in Fig. 1 shows that the empirical correlation matrix actually presents a set of eigenvalues outside of the bulk of the distribution. To check the hypothesis that these eigenvalues may be attributed to specialist behavior, we have simulated the correlation matrix of a system in which suspects are pure specialists. Here we use the term “specialists” to indicate suspects who explore only one specific group of types of crimes, which is identified, in the simulations, by a chapter of the penal code. In our simulations, each suspect is initially associated with a randomly selected type of crime, the probability that a type of crime is selected being proportional to the frequency of the type of crime in the original database. The first type of crime allows one to associate each suspect with a specific chapter of the penal code. So, once the first type of crime is selected, each suspect will continue to randomly explore types of crimes belonging to the chapter of that first type of crime until a number of types of crimes equal to the total number of types of crimes alleged for the suspect in the original database is reached. Again, the types of crimes are selected randomly within a group of types of crimes by setting the probability that a type of crime is selected as proportional to the frequency of that type of crime in the original database. This approach allows us, on average, to preserve the heterogeneity of both types of crimes and suspects. The density of eigenvalues of the correlation matrix of 100 realizations of the simulated database of specialists is reported in the bottom panel of Fig. 1. The largest eigenvalue has an average value of 5.51, which is significantly smaller than the largest eigenvalue of the empirical correlation matrix (λ_M_ = 11.26). The eigenvector components (already for the eigenvector associated with the first eigenvalue) are organized in different groups of types of crimes which are belonging to the same penal chapter and are each characterized by components of the same sign and absolute value decreasing as a function of the frequency of the type of crime (see the panel of Fig. 2 referring to specialists' simulations) In summary, the spectral analysis of the correlation matrix of types of crimes supports the presence of both generalist and specialist suspects in the database.

The simulation of the pure specialists hypothesizes that each group of types of crimes is defined by the corresponding chapter of the penal code. We acknowledge that this is a simplifying assumption and therefore, before we perform the following analyses we look for an approach allowing us to detect clusters of types of crimes directly from real data by using an unsupervised clustering procedure based on network theory.

We start from the bipartite network of types of crimes and suspects. The basic information characterizing the network can be summarized as follows. The number of suspects is 336,069 and the number of types of crimes is 376. In the bipartite network we count 1,078,908 links, it is therefore a quite sparse and heterogeneous bipartite network. The degree of types of crimes ranges from the minimum values of 1 to the maximum value of 81,532. The average value is 2,869 and its standard deviation is 7,206. The degree of suspects also covers a broad range starting from 1 and ending to 159. In the case of suspects, the average degree is 5.51 and its standard deviation is 17.3. Starting from the bipartite network we obtained the projected network of types of crimes as follows. We constructed a projected network of types of crimes by linking two types of crimes when they have been both associated with at least one suspect and we weighted the link as the number of suspects implicated in both crimes. In this way, we obtained a network of 376 crimes connected by 41,556 links in a single large component. Such a network is an almost complete network (in fact a complete network would present 70,500 links).

On this weighted projected network we performed a community search with the Infomap algorithm [26], which is a successful and accurate community detection algorithm [27]. Unfortunately, the algorithm failed to partition the system. The failure was probably due to the fact that the projected network is an almost complete network and community detection is notoriously difficult in almost complete networks. To overcome this difficulty we decided to filter the weighted projected network of types of crimes by selecting only those links presenting an excess (or over-expression) of co-occurrence of suspects while properly taking into account the heterogeneity of both types of crimes and suspects. Specifically, we select the links by adapting a recently proposed method, which is constructing statistically validated networks [28] in heterogeneous complex systems.

### Statistically Validated Networks

To take into account the presence in the database of suspects who were implicated in only one type of crime and suspects that were implicated in many types of crimes, the database has been decomposed into several subsets of data with homogeneous profiles of suspects. Specifically, the first subset *S_1_* of the database includes all of the suspects who were suspected of 1 type of crime, the second subset *S_2_* included all the suspects who were suspected of exactly 2 different types of crimes, and so on. Each subset *S_f_* is therefore identified by the common number *f* of different types of crimes alleged to each suspect in the subset. Altogether we consider n_s_ = 159 different subsets *S_f_*. By construction, each suspect can be present in only one subset, while a given type of crime can potentially be present in all of the different subsets.

The heterogeneity of crime types is still apparent within each subset {S_f_}. Indeed the number of suspects for type of crime varies a lot across different crime types, in spite of the homogeneity of suspects in the subset. For each subset, to properly take into account the heterogeneity of the types of crimes, we set a link between two types of crimes, when the suspicion of two types of crimes has been directed to the same suspects with an occurrence that cannot be explained under a null hypothesis of random co-occurrence of types of crimes. The appropriate null hypothesis is constructed in the following way. Let us consider two types of crimes, say A and B, and a specific subset {*S_f_*}. Let us call N_A_^f^ the number of suspects in {*S_f_*} who were suspected of type of crime A, and N_B_^f^ the number of suspects in {S_f_} suspected of type of crime B. Let us call N_AB_^f^ the number of suspects in {*S_f_*} who were suspected of both types of crimes, A and B. Under the null hypothesis of random co-occurrence, the probability of observing X co-occurrences is given by the hypergeometric distribution
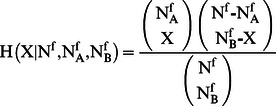
(2)where N^f^ is the total number of suspects in the subset {S_f_}. By using this distribution one can associate a *p*-value with the observed number N_AB_^f^ of co-occurrences, that is



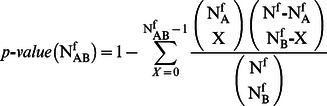
(3)This null hypothesis explicitly takes into account the heterogeneity of both the types of crimes, A and B, by conditioning the probability of co-occurrence to the two occurrences N_A_^f^ and N_B_^f^. We can therefore choose a statistical threshold and assign a link to only those pairs of crime types that are characterized by a p-value lower than the selected threshold. In the present study, we have chosen a statistical threshold of 0.01. To build a weighted network of types of crimes based on the excess of co-occurrence, we compute the p-value associated with all pairs of types of crimes in each subset {*S_f_*}. We therefore perform a multiple comparison involving all pairs of types of crimes present in each subset of the database. In a statistical test performing multiple comparisons, the statistical threshold needs to be properly redefined with respect to the univariate case. Here we use the False Discovery Rate (FDR) method to modify the univariate statistical threshold u_t_ = 0.01. The FDR method prescribes that the threshold u_t_ = 0.01 is initially divided by the total number of tested hypotheses T. Then all the calculated *p*-values are sorted in increasing order (p_1_<p_2_<…<p_T_), and the FDR threshold σ_FDR_ is set equal to p_q_, where q is the largest k such that p_k_<k u_t/_T. In the present case, the total number of tested hypotheses is the sum over all the subsets {S_f_} of the total number of pairs of types of crimes with at least one person suspected of both types of crimes in {*S_f_*}. Specifically, the total number of tested hypotheses is 831,944, and σ_FDR_ = 0.000071. Each calculated *p*-value is compared with the threshold σ_FDR_, and a link between two types of crimes is set if the co-occurrence *p*-value is smaller than σ_FDR_ in at least one subset {S_f_} of the original database, while the weight of the link is set equal to the total number of subsets in which such a statistically significant excess of co-occurrence is detected. The resulting network is called an FDR network after [28]. The FDR network of types of crimes includes 295 types of crimes connected by 1107 weighted links, and the weight of the links ranges between 1 and 38. The subset of suspects which contributes the most to the formation of links in the network is the subset {S_4_} of suspects with only 4 types of crimes in their records (362 links), followed by subsets {S_5_} and {S_7_} (351 links each) and {S_3_} (349 links). The subset with the smallest number of types of crimes per suspect, {S_2_}, contributes to 248 links. Finally, no subset of suspects with more than 50 types of crimes contributes to the statistical validation of a single link (see Table S1 for details).

## Results and Discussion

### Clusters of Types of Crimes

**Figure 3:**
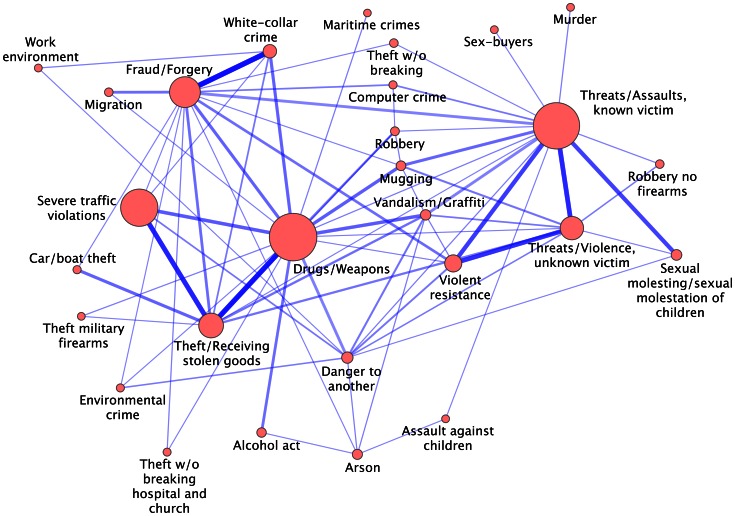
Interrelations of clusters of the FDR statistically validated network of types of crimes. The weight of a link between any two clusters is a monotonic increasing function of the sum of all the weights of links bridging types of crimes of the two clusters. The size of the node associated with each cluster of types of crimes is proportional to the number of suspects involved (see Table 1) in that cluster.

The FDR statistically validated network is a network which is much more sparse than the original projected network. In fact, it has 295 nodes and 1,107 links. By applying the Infomap algorithm [26] the FDR is partitioned in several clusters. Specifically, the algorithm revealed 28 clusters of types of crimes of sizes ranging between 2 and 39. Fig. 3 shows the interrelations of clusters of the FDR network. A link between two clusters is set if at least one significant co-occurrence is detected between a type of crime belonging to the first cluster and a type of crime belonging to the second one. In the figure, the size of a node is a linear function of the number of suspects who explored the corresponding cluster, and the weight of a link between two clusters is a monotonic increasing function of the sum of all the weights of links bridging types of crimes of the two clusters in the FDR network. Each cluster of types of crimes has been characterized in terms of the types of crimes it includes and according to the demographic information associated with suspects who were implicated in one or more types of crimes in the cluster. Demographic information includes gender and year of birth of suspects. We have grouped the years of birth into four categories, 1903–1948, 1949–1962, 1963–1973 and 1974–1987, such that the number of suspects does not vary a lot across the different categories. In Table 1, we report information about the number of crime types (2nd column), number of criminal instances (3rd column), and number of suspects (5th column) belonging to each cluster (labeled in Column 1) detected with Infomap in the FDR network of types of crimes. On Columns 4, 6, and 7 of Table 1 we report the results of the characterization analysis of all the clusters as performed according to the method described in Ref. [29]. Unless specifically indicated, each entry of the 4th, 6th, and 7th column of the Table represents a statistically validated (*p*-value smaller than 1% after correction for multiple hypothesis testing) over-representation of the displayed attributes in the corresponding cluster. The complete list of types of crimes belonging to each cluster is provided in Table S2.

**Table 1:** Characterization of clusters in the FDR network of crime types.

Cluster	# Crimetypes	#Instances	Code Chapter(# of crime types)	#Suspects	Birth Year	Gender
Threats/Assaults, known victim	39	390,483	Ch4(14);Ch3(15)	121,207	1949–1962; 1963–1973	Male
Drugs/Weapons	30	450,435	Drugs(10); Weapons Knives Acts(5)	125,011	1974–1987	Female
Theft	38	223,676	Ch8(34)	53,614	1963–1973; 1974–1987	Male
Fraud/Forgery	34	159,965	Ch9(16); Ch10(6); Ch14(6)	72,602	1949–1962; 1963–1973	Female
White-collar crimes	18	35,299	Tax offences(11); Ch11(5)	18,466	1903–1948; 1949–1962; 1963–1973	Male
Violent resistance	6	68,959	Ch17(6)	29,827	1963–1973; 1974–1987	Male
Severe traffic violations	7	335,278	Road Traffic Act(5)	92,879	1903–1948; 1949–1962; 1963–1973	Male
Threats/Violence, unknown victim	11	80,774	Ch3(9)	49,319	1963–1973; 1974–1987	Male
Sexual molestation of children/Sexual molestation	14	14,121	Ch6(13)	9,675	1903–1948; 1949–1962	Male
Vandalism/Graffiti	5	14,726	Ch12(4)	8,834	1974–1987	Male
Environmental crime	12	2,113	Environmental Code(12)	1,533	1903–1948; 1949–1962	Male
Alcohol	7	7,473	Alcohol Act(6)	5,842	1949–1962	Male
Mugging	7	10,808	Ch8(7)	6,646	1974–1987	Male
Danger to another	8	14,280	–	11,802	1974–1987	Male
Car/Boat theft	3	3,065	Ch8(3)^1^	1,804	1963–1973; 1974–1987	Male
Robbery	10	5,707	Ch8(10)	3,889	1963–1973; 1974–1987	Male
Migration	7	3,631	Aliens act(4)	3,152	1963–1973; 1974–1987	Female
Arson	4	9,194	Ch13(3)	7,936	1903–1948	–
Computer crimes	3	2,212	–	1,887	1903–1948; 1949–1962	Male
Work environment	5	857	–	751	1903–1948; 1949–1962	Male
Sex-buyers	4	861	–	654	1949–1962; 1963–1973	Male
Murder	5	809	Ch3(5)	735	1974–1987	Male
Theft military firearms	4	561	Ch8(4) 1	464	1963–1973; 1974–1987	Male
Robbery, no firearms	3	4,094	Ch8(3) 1	3,064	1963–1973; 1974–1987	Male
Assault against children	4	785	Ch3(4) 1	713	1949–1962; 1963–1973	Male
Theft w/o breaking	3	3,765	–	3,223	1963–1973; 1974–1987	Male
Maritime crimes	2	77	Road Traffic Act(2) 1	64	–	Male
Theft w/o breaking hospital/church	2	1,770	Ch8(2) 1	1,283	1949–1962; 1963–1973	–

1 Not statistically significant as the cluster is too small with respect to the total number of crimes with that attribute.

The clusters detected with our network-based method arrange types of crimes in a partition that shows seven clusters characterized by an over-expression of a unique chapter of the penal code and four clusters with an over-expression of more than two distinct chapters. Some of the other twelve clusters show over-expression of Chapters 3 (Crimes against life and health) and 8 (Theft, robbery, and other crimes of stealing) and of traffic violations, whereas only five clusters are not characterized by over-expression of at least one chapter of the penal code. The network of clusters shown in Fig. 3 allows us to discuss some relevant connections between clusters. A tight connection is observed between the cluster *Threats/Assaults, known victim* and *Sexual molestation of children/Sexual molestation*, indicating the presence of a pattern that relates types of crimes of assault in which the suspect is acquainted with the victim to sexual offenses against underage victims. *Cluster Threats/Assaults, known victim* is also strongly connected to the cluster *Violent resistance*, which is the cluster of violence against public servants, and cluster *Violent resistance*, in turn, is connected with cluster *Threats/Violence, unknown victim*, which includes types of crimes of assault in which the suspect is not acquainted with the victim. The loop is finally closed by the connection between cluster *Threats/Violence*, *unknown victim*, and the cluster *Threats/Assaults, known victim*. An expected connection is observed between the cluster *Fraud/Forgery* and the cluster *White-collar crime*. Another interesting loop cycle is the one that involves the clusters *Drugs/Weapons*, *Theft* and *Severe traffic violations*.

### Generalist and Specialist Behavior

In this section, we discuss the generalist behavior of suspects belonging to different categories of gender and year of birth. The generalist behavior is investigated by comparing the properties of the real system with the properties of a system obtained by randomly shuffling the original database (pure generalist hypothesis). Specifically, for each category of suspects, we count the total number of clusters of types of crimes found in the FDR network that each suspect explores during their criminal activity, both for real and shuffled data. Then we count the fraction of suspects who explore only one cluster, the fraction of suspects who explore 2 clusters, and so on. People who were suspected of only one type of crime are forced to explore only one cluster both in the real and the shuffled database. Therefore this group of suspects is removed from the present analysis. The suspects explore, on average, fewer clusters than are observed for pure generalists (shuffled data), and this result is stable across the different categories of suspects. In Table 2, we report the mean value and the standard deviation of the number of explored clusters of types of crimes both for real data (second and third column) and for the pure generalist simulation (fourth and fifth column). Results for generalists average over 1,000 independent shuffled replicas of the empirical database. The mean value of real data is always significantly less than the mean value of simulated data, indicating the presence of a certain degree of specialization. For all reported cases the *p*-value associated with a null hypothesis that both empirical and simulated mean values come from the same distribution is always less than 10^−16^ when tested with a Welch's *t*-test.

**Table 2:** Mean value of the number of visited clusters of crime types.

Category	mean	std	mean	std	P(1)	P(1)
	(data)	(data)	(simul.)	(simul.)	(data)	(simul.)
All	2.68	1.87	3.73	2.55	0.264	0.06
1903–1948	2.07	1.31	3.13	1.99	0.385	0.0746
1949–1962	2.64	1.83	3.92	2.75	0.269	0.0555
1963–1973	2.83	1.96	3.92	2.72	0.234	0.0562
1974–1987	3.01	2.05	3.78	2.47	0.2	0.0576
Male	2.83	1.95	3.88	2.63	0.232	0.0554
Female	2.07	1.34	3.12	2.09	0.391	0.0784

Our results, summarized in Table 2, show that specialization is more pronounced in women than in men both in absolute and relative terms with respect to the pure generalist case. In fact, the relative decrease in the mean value of the number of explored clusters with respect to the one observed for the pure generalist case is 27% for men and 34% for women. Specialization is also more pronounced in older than in younger suspects, again both in absolute and relative terms. In fact, the relative decrease in the mean value of the category of youngest suspects (1974–1987) is 20%, whereas it is 34% for the oldest suspects (1903–1948).

In Table 2 we also report for real and simulated data the fraction of suspects who explored one cluster P(1). This subset of suspects can be considered as the subset of pure specialists limiting their criminal action to only types of crimes belonging to a single cluster. The fraction of pure specialists in real data is significantly higher than the amount expected in the pure generalist case. The standard deviation of P(1), over the 1,000 independent shufflings of the database, in the generalist case is always less than 0.0014. Results show that real data present a significant number of pure specialists. It is worth remembering that this fraction does not include individuals suspected of only one type of crime. Again, Table 2 shows that women have a higher proportion of pure specialists than men, and that the degree of pure specialization increases with age.

### Conclusions

The study of criminal specialization is complicated by the fact that classifications of crime types are to a considerable extent ad hoc derivations from the penal code rather than empirically based on criminals’ behavior. We used concepts and tools from network science to empirically detect clusters of types of crimes and relationships among them. Several empirically derived indicators support the conclusion that there is a core of clusters that connect “traditional” types of crimes, including violent crimes, drug related crimes, thefts, burglaries, and frauds of different types. Close to this core, we also find financial crime, traffic violations, and organized robbery. On the other hand, the periphery of the crime types network contains a heterogeneous set of types of crimes, including “modern” crimes such as environmental violations, but also sex crimes. In parallel, we observe a non-trivial pattern of specialization across time and gender. In general, women are implicated in types of crimes classified in fewer clusters, and tend to be more specialized than men. We also find that older persons are the most specialized suspects. This can be due to three different or combined factors: (i) suspects tend to specialize over time; (ii) there is a group of specialized individuals who remain in crime, while the generalists distance themselves from criminal activity; and (iii) there is a cohort effect such that the younger generation tends to consist of generalists while the older generation consists of specialists.

The spectral analysis of the correlation matrix of types of crimes supports the idea that there is a limited number of crime specialists and only minor specialization in a few select types of offenses [1]. The analysis of clusters of types of crimes in the FDR network shows that types of crimes of a similar nature are grouped in large clusters. This observation suggests that some suspects tend to concentrate their criminal activity into the major crime categories [30] obtained from the unsupervised classification based on network theory. However, we also observed small groups of quite homogeneous types of crimes that indicate the presence of specialization at the level of minor categories of types of crimes as well. Prominent examples are the cluster *Sexual molestation of children/Sexual molestation*, *Environmental crime*, *Murder,* and *Robbery*.

The results reported in Table 2 also indicate that the little specialization that still exists occurs after adolescence and increases with criminal career progression [30], [31]. More specific results about the relation between criminal-career progression and specialization are reported in Fig. S3. We also observe that specialization in women is higher than in men. Our results also suggest that age-group specialization is related to the category of crime. Indeed, in Table 1 one may observe that some clusters, namely *Drugs/Weapons*, *Vandalism/Graffiti*, *Mugging*, and *Murder* present a statistically significant over-expression of young people among the suspects of these types of crimes. At the other extreme, the cluster *Arson* presents an overrepresentation of old people among the suspects. Specialization also seems to be related to the type of victim, like underage victims in cluster *Sexual molestation of children/Sexual molestation*, to the use of firearms (*Drugs/Weapons* versus *Robbery* and *Robbery, no firearms*), and to the level of organization required (*Robbery* of banks, post offices or security vans).

In conclusion, the present analysis contributes to the understanding of the interrelationships among types of crimes, allows for an evaluation of the degree of generalism and specialism of suspects present in the database, and reveals different types of specialization that can be characterized by the attributes of suspects and victims, by the means used, and by the types of crimes. Some criminological theories imply specialization and others do not. Our results show specialization for certain offenses and certain types of offenders but not for others. We suggest that different types of offenses and offenders can be modeled and explained by different theoretical approaches depending on the degree of specialization associated with the criminal activity of interest.

## Supporting Information

Figure S1 Heterogeneity of types of crimes and suspects. The top panel describes the heterogeneity of types of crimes in terms of the number of different suspects per type of crime. Type of crimes are ordered in decreasing order of number of suspects per type of crime. The bottom panel describes the heterogeneity of suspects, as the number of suspects (vertical axis) alleged of a number of different types of crimes (horizontal axis).
(TIFF)

Figure S2 Comparison of the eigenvalues of the correlation matrix of types of crimes obtained by including all suspects (black circles) or suspects suspected of two or more crime types (red circles). The y-axis gives the value of the eigenvalue whereas the x-axis gives its rank.
(TIFF)

Figure S3 Criminal specialization as a function of criminal career progression. As a proxy of the criminal career progression, we consider the number of types of crimes alleged to each suspect (t in the horizontal axis) in the past. The degree of specialization is calculated as the fraction of suspects, at a level t of career progression, who explore, when suspected of crime type t+1, the same cluster they explored through type of crime t (P[C(t+1) = C(t)] in the vertical axis). The size and internal pattern of circles that have been used to display data points in the figure, change from left to right, in order to provide a guide to the eye for the decreasing statistics (number of suspects) that has been used to calculate the probability at increasing values of t.
(TIFF)

Table S1 List of validated links in the FDR crime types network. The validation information is provided for each link and for each subset of suspects contributing to the validation of each link.
(XLSX)

Table S2 List of types of crimes belonging to each cluster in the network.
(XLSX)

## References

[1] Loeber R, Farrington DP, eds. (1998) Serious & Violent Juvenile Offenders. Risk Factors and Successful Interventions, Thousand Oaks: Sage.

[2] Tracy PE, Kempf-Leonard K (1996) Continuity and Discontinuity in Criminal Careers. New York: Plenum Press.

[3] Osgood DW, Schreck CJ (2007) A new method for studying the extent, stability, and predictors of individual specialization in violence. Criminology 45: 273–312.

[4] Piquero AR, Farrington DP, Blumstein A (2007) Key Issues in Criminal Career Research. Cambridge: Cambridge University Press.

[5] Bursik RJ Jr (1980) The Dynamics of Specialization in Juvenile Offenses. Social Forces 58: 851–864.

[6] Kempf K (1987) Specialization and the criminal career. Criminology 25: 399–420.

[7] Piquero A (2000) Frequency, Specialization, and Violence in Offending Careers. J. of Research in Crime and Delinquency 37: 392–418.

[8] Gottfredson MR, Hirschi T (1990) General Theory of Crime. Stanford University Press.

[9] Hirschi T (1969) Causes of Delinquency, University of California Press.

[10] Kornhauser RR (1978) Social Sources of Delinquency - An Appraisal of Analytic Models, University of Chicago Press.

[11] Damasio AR (1995) Descartes’ Error: Emotion, Reason, and Human Brain, New York: Avon Books.

[12] Nylen TC (1999) Frontal Lobe Function: Mr. Phineas Gage’s Famous Injury, J. of Neuropsychiatry and Clinical Neurosciences 11(2): 280–281. 10.1176/jnp.11.2.280 10334002

[13] Virkkunen M, Linnoila M (1993) Brain serotonin, Type II alcoholism and impulsive violence. J. of Studies on Alcohol, Suppl 11: 163–169. 10.15288/jsas.1993.s11.163 8410958

[14] Alm PO, Af Klinteberg B, Humble K, Leppert J, Sörensen S, et al (1996) Criminality and psychopathy as related to thyroid activity in former juvenile delinquents. Acta Psychiatrica Scandinavica 94: 112–117. 8883572 10.1111/j.1600-0447.1996.tb09834.x

[15] Bowes L, Arseneault L, Maughan B, Taylor A, Caspi A, et al (2009) School, Neighborhood, and Family Factors Are Associated With Children's Bullying Involvement: A Nationally Representative Longitudinal Study. J. of the Am. Academy of Child & Adolescent Psychiatry 48: 545–553. 10.1097/CHI.0b013e31819cb017 PMC4231780 19325496

[16] Wolfgang ME, Ferracuti F (1967) The subculture of violence: Toward an integrated theory of criminality, Tavistock, London.

[17] Sutherland EH (1949) White Collar Crime: The Uncut Version, New Heaven Yale University Press.

[18] Sutherland EH, Cressey DR, Luckenbill DF (1992) Principles of criminology. Rowman & Littlefield.

[19] Cloward RA, Ohlin LE (1960) Delinquency and opportunity, Free Press Corporation.

[20] Foucault M (1975) Surveiller et punir: Naissance de la prison, Paris: Gallimard.

[21] Newman M (2010) Networks: An Introduction, Oxford: Oxford University Press.

[22] Sarnecki J (2001) Delinquent networks, Studies in Criminology, Cambridge: Cambridge University Press.

[23] von Dijk J (2008) The World of Crime: Breaking the Silence on Problems of Security, Justice and Development Across the World. Sage Publications, Inc.

[24] Laloux L, Cizeau P, Bouchaud J-P, Potters M (1999) Noise dressing of financial correlation matrices. Phys Revi Lett 83: 1467–1470.

[25] Plerou V, Gopikrishnan P, Rosenow B, Amaral LAN, Stanley HE (1999) Universal and nonuniversal properties of cross correlations in financial time series. Phys Rev Lett 83: 1471–1474.

[26] Rosvall M, Bergstrom CT (2008) Maps of random walks on complex networks reveal community structure. Proc Natl Acad Sci USA 105, 1118–1123. 10.1073/pnas.0706851105 PMC2234100 18216267

[27] Fortunato S (2010) Community detection in graphs. Physics Reports 486: 75–174.

[28] Tumminello M, Miccichè S, Lillo F, Piilo J, Mantegna RN (2011) Statistically validated networks in bipartite complex systems, PLoS ONE 6 (3) e17994 10.1371/journal.pone.0017994 PMC3069038 21483858

[29] Tumminello M, et al.. (2011) Community characterization of heterogeneous complex systems. J Stat Mech-Theory Exp P01019.

[30] Blumstein A, Cohen J, Roth JA, Visher CA, eds. (1986) Criminal Careers and ‘Career Criminals’ Volume 1. Washington DC: National Academy Press.

[31] Piquero A, Patern Oster R, Mazerolle P, Brame R, Dean CW (1999) Onset Age and Offense Specialization. J. of Research in Crime and Delinquency 36: 275–299.

