# Supplementary figures and images for: The Phenomenology of Specialization of Criminal Suspects
Source: PLoS One. 2013 May 15;8(5):e64703. doi: 10.1371/journal.pone.0064703 (PMC3654921; doi:10.1371/journal.pone.0064703)

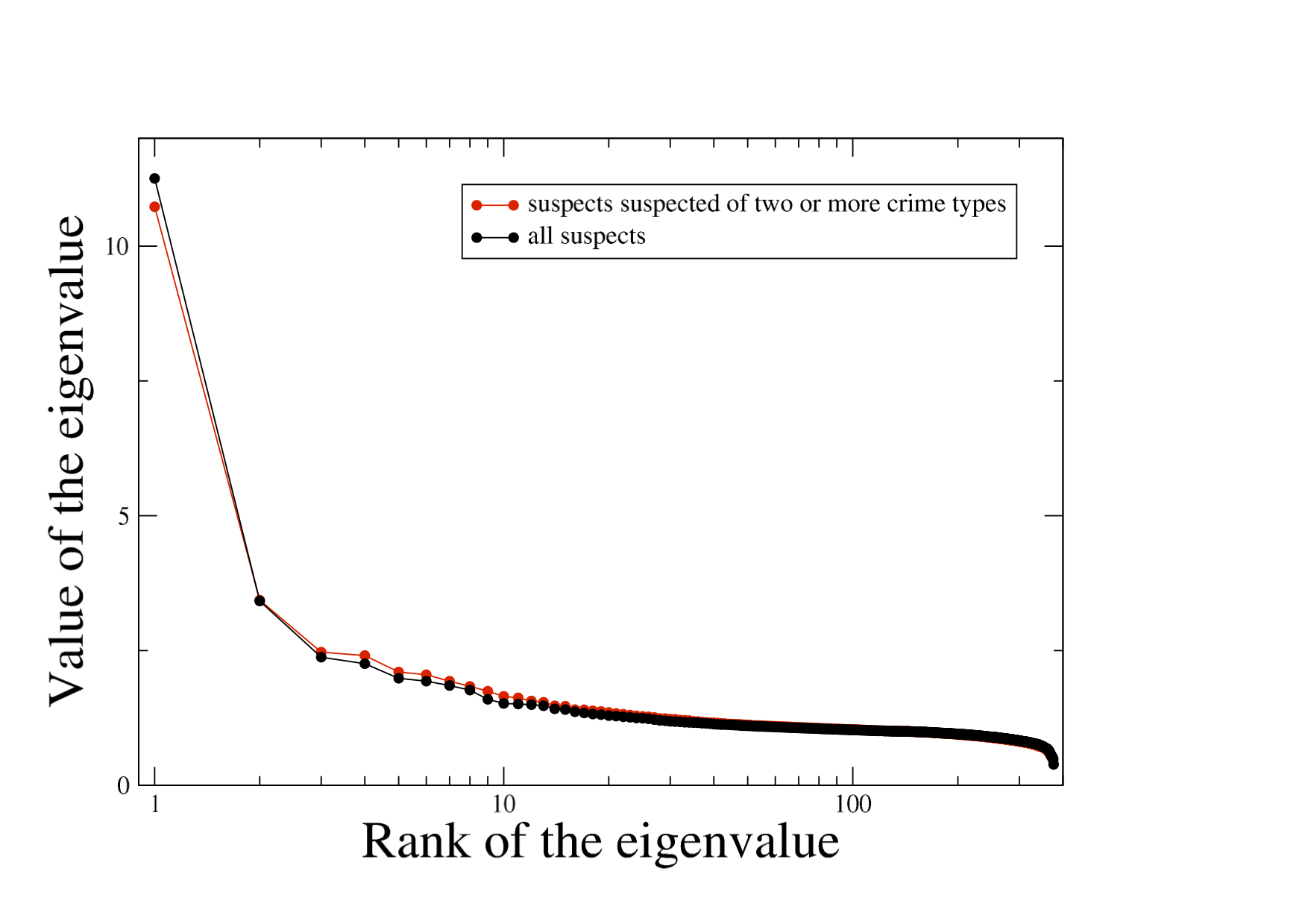

Supplement: Figure S2 — Comparison of the eigenvalues of the correlation matrix of types of crimes obtained by including all suspects (black circles) or suspects suspected of two or more crime types (red circles). The y-axis gives the value of the eigenvalue whereas the x-axis gives its rank. (TIFF) [file pone.0064703.s002.tiff]

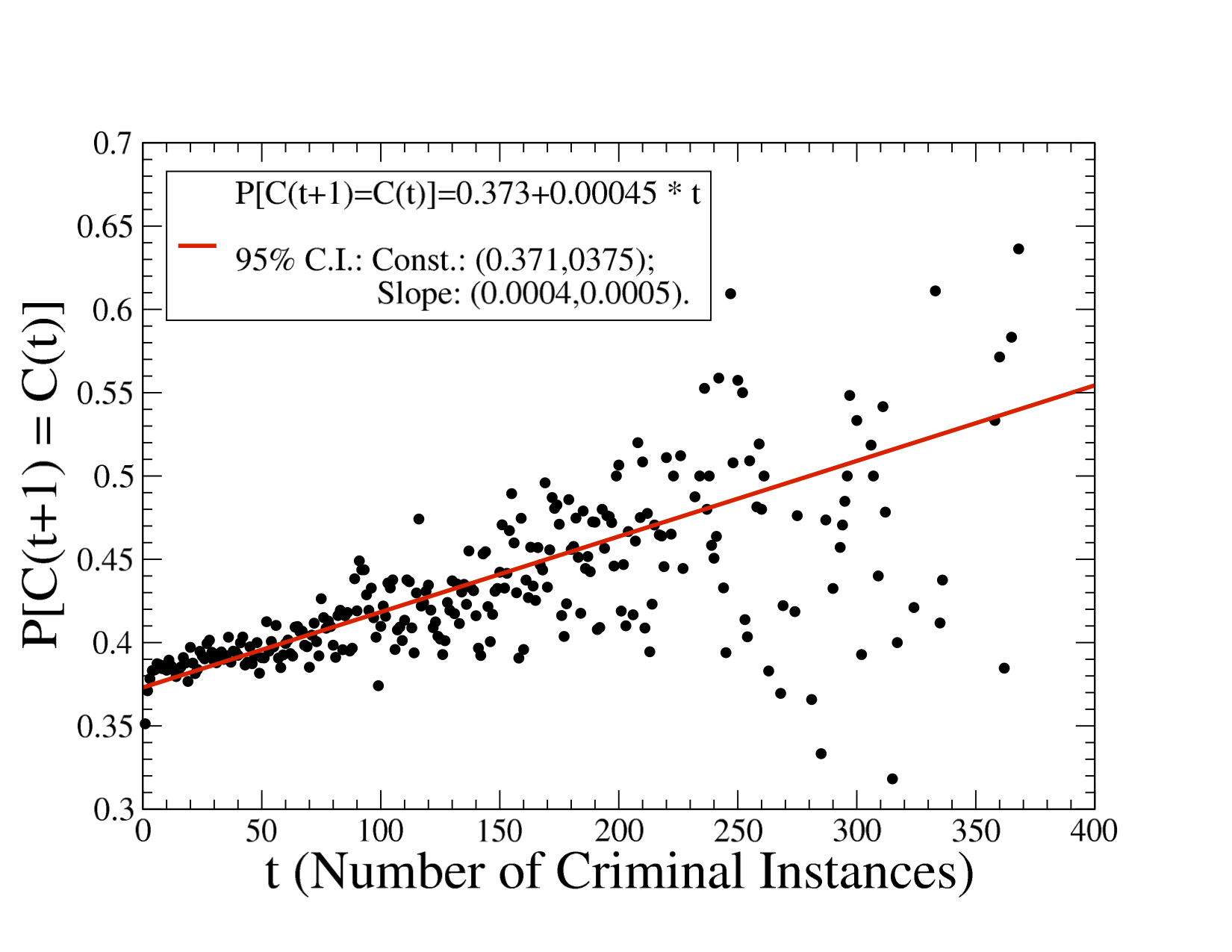

Supplement: Figure S3 — Criminal specialization as a function of criminal career progression. As a proxy of the criminal career progression, we consider the number of types of crimes alleged to each suspect (t in the horizontal axis) in the past. The degree of specialization is calculated as the fraction of suspects, at a level t of career progression, who explore, when suspected of crime type t+1, the same cluster they explored through type of crime t (P[C(t+1) = C(t)] in the vertical axis). The size and internal pattern of circles that have been used to display data points in the figure, change from left to right, in order to provide a guide to the eye for the decreasing statistics (number of suspects) that has been used to calculate the probability at increasing values of t. (TIFF) [file pone.0064703.s003.tiff]
